# Association of Lipoprotein(a)-Associated Mortality and the Estimated Glomerular Filtration Rate Level in Patients Undergoing Coronary Angiography: A 51,500 Cohort Study

**DOI:** 10.3389/fcvm.2021.747120

**Published:** 2021-11-17

**Authors:** Zhidong Huang, Yanfang Yang, Jin Lu, Jingjing Liang, Yibo He, Yaren Yu, Haozhang Huang, Qiang Li, Bo Wang, Shanggang Li, Zelin Yan, Danyuan Xu, Yong Liu, Kaihong Chen, Zhigang Huang, Jindong Ni, Jin Liu, Liling Chen, Shiqun Chen

**Affiliations:** ^1^Department of Cardiology, Guangdong Provincial Key Laboratory of Coronary Heart Disease Prevention, Guangdong Cardiovascular Institute, Guangdong Provincial People's Hospital, Guangdong Academy of Medical Sciences, Guangzhou, China; ^2^The Graduate School of Clinical Medicine, Fujian Medical University, Fuzhou, China; ^3^Department of Cardiology, Longyan First Affiliated Hospital of Fujian Medical University, Longyan, China; ^4^The Second School of Clinical Medicine, Southern Medical University, Guangzhou, China; ^5^Department of Cardiology, The First People's Hospital of Foshan, Foshan, China; ^6^Department of Public Health, Guangdong Medical University, Dongguan, China

**Keywords:** lipoprotein(a), estimated glomerular filtration rate, all-cause mortality, coronary angiography, renal function

## Abstract

**Background:** High lipoprotein(a) is associated with poor prognosis in patients at high risk for cardiovascular disease. Renal function based on the estimated glomerular filtration rate (eGFR) is a potential risk factor for the change of lipoprotein(a). However, the regulatory effect of eGFR stratification on lipoprotein(a)-associated mortality has not been adequately addressed.

**Methods:** 51,500 patients who underwent coronary angiography (CAG) or percutaneous coronary intervention (PCI) were included from the Cardiorenal ImprovemeNt (CIN) study (ClinicalTrials.gov NCT04407936). These patients were grouped according to lipoprotein(a) quartiles (Q1–Q4) stratified by eGFR categories (<60 and ≥60 mL/min/1.73m^2^). Cox regression models were used to estimate hazard ratios (HR) for mortality across combined eGFR and lipoprotein(a) categories.

**Results:** The mean age of the study population was 62.3 ± 10.6 years, 31.3% were female (*n* = 16,112). During a median follow-up of 5.0 years (interquartile range: 3.0–7.6 years), 13.0% (*n* = 6,695) of patients died. Compared with lipoprotein(a) Q1, lipoprotein(a) Q2–Q4 was associated with 10% increased adjusted risk of death in all patients (HR: 1.10 [95% CI: 1.03–1.17]), and was strongly associated with about 23% increased adjusted risk of death in patients with eGFR <60 mL/min/1.73m^2^ (HR: 1.23 [95% CI: 1.08–1.39]), while such association was not significant in patients with eGFR ≥60 mL/min/1.73m^2^ (HR: 1.05 [95% CI: 0.97–1.13]). P for interaction between lipoprotein(a) (Q1 vs. Q2–Q4) and eGFR (≥60 vs. eGFR <60 mL/min/1.73m^2^) on all-cause mortality was 0.019.

**Conclusions:** Elevated lipoprotein(a) was associated with increased risk of all-cause mortality and such an association was modified by the baseline eGFR in CAG patients. More attention should be paid to the patients with reduced eGFR and elevated lipoprotein(a), and the appropriate lipoprotein(a) intervention is required.

## Introduction

Lipoprotein(a) is a low-density lipoprotein (LDL) particle covalently bound to a large glycoprotein, apolipoprotein(a) [apo(a)] (1). Recent advances have once again thrust lipoprotein(a) into the clinical spotlight that large evidence in genetic and epidemiology verified that high lipoprotein(a) level was an independent risk factor for morbidity and mortality of atherosclerotic cardiovascular disease (2–6). Many pathogenic pathways are activated in this complex process including pro-atherogenic, pro-thrombotic, and pro-oxidative properties (4). Lipoprotein(a) has been recommended to identify high risk patients with coronary artery disease (CAD) by the last European and American cholesterol management guidelines (7, 8). Several potent and specific therapies including proprotein convertase subtilisin/kexin type 9 (PCSK9) inhibitors and antisense oligonucleotides, are currently being developed clinically to lower plasma lipoprotein(a) concentrations (9, 10).

The metabolism of lipoprotein(a), though still not well-understood, appears to be related to renal function. The reason for increased lipoprotein(a) concentrations in renal dysfunction patients probably derives from the decreased clearance or an increased synthesis in the liver caused by the proteinuria (11, 12). Understanding the potential interplay of lipoprotein(a) and renal function may allow optimal personalized treatment of patients with or without multiple risk factors. To date, however, limited data exist on the association between lipoprotein(a) and the prognosis in patients with different degrees of renal dysfunction.

Given that level of the estimated glomerular filtration rate (eGFR) could reflect the degree of renal insufficiency (13, 14). Our objective was to examine the relationship between lipoprotein(a) and all-cause mortality across categories of eGFR.

## Method

### Data Sources and Study Population

The Cardiorenal ImprovemeNt (CIN) study is a single-center, retrospective and observational cohort study, enrolling 88,938 consecutive patients undergoing coronary angiography (CAG) or percutaneous coronary intervention (PCI) in Guangdong Provincial People's Hospital, Guangdong, China, hospitalized in between January 2007 to December 2018 (ClinicalTrials.gov NCT04407936). PCI was performed following standard clinical practice guidelines. Exclusive criteria included: (a) patients without data of baseline lipoprotein(a) (*n* = 13,848); (b) patients without data of baseline eGFR (*n* = 15,165); c) and patients without follow-up data (*n* = 8,425). These data were missing randomly. Eventually, 51,500 patients were included (Supplementary Figure 1).

### Baseline Data Collection

From January 2007 to December 2018, data were extracted from the electronic clinical management records system of the Guangdong Provincial People's Hospital. We had access to all primary and secondary care records. The baseline information included demographic characteristics, coexisting conditions, laboratory examinations, and medications at discharge. Blood samples except lipid profiles were collected at admission or before CAG and PCI. The lipoprotein(a) was measured by an overnight fasting venous blood sample. The death of patients after discharge were recorded by the attending physician or a trained research assistant at the follow-up.

### Measurement of Lipoprotein(a)

Lipoprotein(a) mass was measured by the latex-enhanced immunoturbidimetric assay using an automatic biochemical analyzer (Beckman AU5800, USA). The detection principles were as follows: Denka antibodies which were anti-lipoprotein(a) polyclonal antibodies coupled to latex microparticles react with lipoprotein(a) in the sample to form an antigen/antibody complex leading to agglutination causing turbidity of the reaction mixture. The absorbance of the compound was proportional to the concentration of lipoprotein(a) in the specimen (manufacturer: Beckman Coulter, Brea, California, unit: mg/dL, normal range: <30 mg/dL). Measurements are linear in the range of 5.0–80.0 mg/dL. The intraassay coefficient of variation was ≤ 10%. Danka calibrator samples were used for correction to minimize the impact of apo(a) isoforms.

### Endpoint and Clinical Definition

The primary endpoint was all-cause mortality which was monitored and recorded by trained nurses and research assistants through outpatient interviews and telephones. The level of lipoprotein(a) was quartered into four groups (quartiles Q1:0–8.30 mg/dL, Q2:8.30–15.30 mg/dL, Q3:15.30–32.10 mg/dL, Q4:32.10–522.44 mg/dL). The eGFR was calculated by the Modification of Diet in Renal Disease formula (15), and was divided into 2 categories of eGFR (<60, ≥60 mL/min/1.73m^2^). eGFR[mL/(min·1.73m^2^)] = 186 × [SCr(μmol/L) ×0.011312]^−1.154^ × age^−0.203^(if female ×0.762) (16). Anemia was defined as a hematocrit ≤ 39% (male) or ≤ 36% (female). Congestive heart failure (CHF) was defined as New York Heart Association class > 2 or Killip class > 1 (17). CAD, acute myocardial infarction (AMI), valvular heart disease (VHD), atrial fibrillation (AF), hypertension (HT), and diabetes mellitus (DM) were defined using ICD-10 codes.

### Statistical Analysis

Baseline characteristics are presented as mean ± SD for continuous variables, and proportions for categorical variables. The differences of baseline characteristics between groups were compared using Student *t*-test for continuous variables and chi-square tests for categorical variables. The differences of characteristics in box plot were compared using Kruskal-Wallis. Time-to-event data among groups are presented graphically using Kaplan-Meier curves and compared by the log-rank test. Multivariable Cox regression models were used to estimate hazard ratios for mortality across combined eGFR and lipoprotein(a) categories, or respectively, with adjustment for major covariables including age, gender, PCI, AMI, HT, DM, anemia, stroke, CHF, CAD, VHD, AF, LDL cholesterol (LDL-C), high-density lipoprotein cholesterol (HDL-C), triglycerides (TRIG), angiotensin-converting enzyme inhibitor or angiotensin receptor blocker (ACEI or ARB), beta-blockers; statins. The *p*-value for interactions between categories of eGFR and lipoprotein(a) in all-cause mortality were estimated using the Wald chi-square test. Presented tests were 2-tailed for all, and a *p*-value <0.05 was considered statistically significant. All statistical analyses were performed using *R* (ver. 4.0.3).

## Result

There were 51,500 patients included in total in this study. The mean age of the study population was 62.3 ± 10.6 years, 31.3% were female. There were significant differences in prevalence of anemia, AMI, CHF, PCI, CAD and the concentration of serum total cholesterol and LDL-C, and use of statins and ACEI or ARB among four groups (Q1, Q2, Q3, and Q4) (Table 1). Compared with patients with eGFR ≥60 mL/min/1.73m^2^, patients with eGFR <60 mL/min/1.73m^2^ were older, and had a higher prevalence of HT, DM and lower LDL-C and HDL-C (Table 1). Baseline characteristics across lipoprotein(a) and eGFR categories was shown in Supplementary Tables 1, 2. There were significant differences between the concentrations of lipoprotein(a) in different eGFR categories (*p* <0.001) (Supplementary Figure 1).

**Table 1:** Baseline characteristics across eGFR and lipoprotein(a) categories.

**Characteristics**		**Lipoprotein(a), mg/dL**				***P*-value**	**eGFR, mL/min/1.73m** ^**2**^		***P*-value**
	**Overall**	**Q1**	**Q2**	**Q3**	**Q4**		**eGFR ≥60**	**eGFR <60**	
	***n* = 51,500**	***n* = 12,818**	***n* = 12,881**	***n* = 12,915**	***n* = 12,886**		***n* = 41,619**	***n* = 9,881**	
**Demographic characteristics**									
Age, year	62.3 ± 10.6	61.5 ± 10.6	62.2 ± 10.7	63.0 ± 10.5	62.7 ± 10.4	<0.001	61.0 ± 10.3	68.0 ± 9.9	<0.001
Female, n (%)	16112 (31.3)	4130 (32.2)	4117 (32.0)	3898 (30.2)	3967 (30.8)	0.001	12784 (30.7)	3328 (33.7)	<0.001
**Medical history**									
AMI, n (%)	7477 (14.5)	1152 (9.0)	1780 (13.8)	2190 (17.0)	2355 (18.3)	<0.001	5733 (13.8)	1744 (17.7)	<0.001
CHF, n (%)	5493 (10.7)	1127 (8.8)	1321 (10.3)	1525 (11.8)	1520 (11.8)	<0.001	3556 (8.6)	1937 (19.6)	<0.001
Anemia, n (%)	15633 (30.6)	3357 (26.4)	3639 (28.5)	4105 (32.1)	4532 (35.5)	<0.001	10850 (26.3)	4783 (48.8)	<0.001
HT, n (%)	26345 (51.2)	6528 (51.0)	6460 (50.2)	6579 (51.0)	6778 (52.7)	0.001	19659 (47.3)	6686 (67.8)	<0.001
DM, n (%)	12026 (23.4)	3152 (24.6)	2844 (22.1)	2997 (23.2)	3033 (23.6)	<0.001	8826 (21.2)	3200 (32.4)	<0.001
PCI, n (%)	25612 (49.7)	5246 (40.9)	6018 (46.7)	6694 (51.8)	7654 (59.4)	<0.001	20013 (48.1)	5599 (56.7)	<0.001
CAD, n (%)	0.68 (0.47)	0.60 (0.49)	0.65 (0.48)	0.70 (0.46)	0.77 (0.42)	<0.001	0.66 (0.47)	0.77 (0.42)	<0.001
VHD, n (%)	8719 (16.9)	2456 (19.2)	2304 (17.9)	2191 (17.0)	1768 (13.7)	<0.001	6963 (16.7)	1756 (17.8)	0.012
AF, n (%)	4318 (8.4)	1328 (10.4)	1172 (9.1)	1058 (8.2)	760 (5.9)	<0.001	3292 (7.9)	1026 (10.4)	<0.001
**Laboratory tests**									
WBC, 10^9^/L	7.79 ± 2.66	7.55 ± 2.53	7.78 ± 2.71	7.89 ± 2.71	7.94 ± 2.67	<0.001	7.65 ± 2.49	8.39 ± 3.23	<0.001
HGB, g/L	133.03 ± 16.90	134.72 ± 16.23	133.89 ± 16.36	132.52 ± 17.27	131.01 ± 17.47	<0.001	134.76 ± 15.48	125.77 ± 20.36	<0.001
TC, mmol/L	4.56 ± 1.17	4.34 ± 1.11	4.51 ± 1.12	4.62 ± 1.16	4.78 ± 1.24	<0.001	4.58 ± 1.16	4.48 ± 1.20	<0.001
TRIG, mmol/L	1.60 ± 1.17	1.84 ± 1.56	1.59 ± 1.15	1.48 ± 0.92	1.51 ± 0.88	<0.001	1.59 ± 1.16	1.66 ± 1.21	<0.001
APOA, g/L	1.13 ± 0.28	1.15 ± 0.28	1.13 ± 0.27	1.12 ± 0.27	1.11 ± 0.28	<0.001	1.14 ± 0.28	1.08 ± 0.27	<0.001
APOB, g/L	0.85 ± 0.24	0.79 ± 0.22	0.84 ± 0.22	0.87 ± 0.24	0.91 ± 0.24	<0.001	0.85 ± 0.23	0.85 ± 0.24	0.005
LDL-C, mmol/L	2.81 ± 0.94	2.56 ± 0.86	2.76 ± 0.90	2.88 ± 0.94	3.02 ± 1.00	<0.001	2.83 ± 0.94	2.73 ± 0.95	<0.001
HDL-C, mmol/L	1.03 ± 0.28	1.02 ± 0.28	1.03 ± 0.27	1.03 ± 0.27	1.03 ± 0.28	<0.001	1.04 ± 0.28	0.99 ± 0.28	<0.001
HbA1c, %	6.38 ± 1.29	6.36 ± 1.28	6.33 ± 1.22	6.38 ± 1.29	6.45 ± 1.38	<0.001	6.33 ± 1.28	6.61 ± 1.33	<0.001
**Medications**									
ACEI or ARB, n (%)	20207 (40.5)	4519 (36.6)	4956 (39.7)	5179 (41.3)	5553 (44.2)	<0.001	16428 (40.5)	3779 (40.0)	0.354
Beta-blockers, n (%)	34676 (69.4)	8194 (66.3)	8456 (67.7)	8752 (69.7)	9274 (73.8)	<0.001	27805 (68.6)	6871 (72.8)	<0.001
Statins, n (%)	39171 (78.4)	9116 (73.7)	9551 (76.5)	9909 (79.0)	10595 (84.3)	<0.001	31466 (77.7)	7705 (81.6)	<0.001

*Values are, n (%) or mean ± SD*.

*AMI, acute myocardial infarction; CHF, congestive heart failure; HT, hypertension; DM, diabetes mellitus; PCI, percutaneous coronary intervention; CAD, coronary artery disease; VHD, valvular heart disease; AF, atrial fibrillation; WBC, white blood cell; HGB, hemoglobin; TC, serum total cholesterol; TRIG, triglycerides; APOA, apolipoprotein A; APOB, apolipoprotein B; LDL-C, low-density lipoprotein cholesterol; HDL-C, high-density lipoprotein cholesterol; HbA1c, glycosylated hemoglobin; ACEI or ARB, angiotensin-converting enzyme inhibitor or angiotensin receptor blocker*.

### Lipoprotein(a), EGFR, and All-Cause Mortality

During a median follow-up of 5.0 years (interquartile range: 3.0–7.6 years), 13% (*n* = 6,695) patients died. Compared with lipoprotein(a) Q1, lipoprotein(a) Q2, Q3, and Q4 were all associated with about 10% increased adjusted risk of death in all patients (Table 2). The cumulative hazard for all-cause mortality across lipoprotein(a) quartiles and eGFR categories shown in Supplementary Figure 2.

**Table 2:** Lipoprotein(a) and eGFR in relation to all-cause mortality, respectively.

**Categories**	**Events (%)**	**Crude**		**Adjusted**	
		**HR (95% CI)**	***P*-value**	**HR (95% CI)**	***P*-value**
**Lipoprotein(a), mg/dL**					
Continuous variable per 10 units		1.002 (1.001–1.003)	<0.001	1.001 (1.000–1.002)	0.010
Quartile					
Q1	1429 (11.15%)	ref		ref	
Q2	1661 (12.89%)	1.11 (1.04–1.20)	0.003	1.08 (1.00–1.16)	0.043
Q3	1813 (14.04%)	1.21 (1.12–1.29)	<0.001	1.10 (1.02–1.19)	0.008
Q4	1839 (14.27%)	1.21 (1.13–1.30)	<0.001	1.11 (1.03–1.19)	0.007
Categories					
Q1	1429 (11.15%)	ref		ref	
Q2–Q4	5313 (13.74%)	1.18 (1.11–1.25)	<0.001	1.10 (1.03–1.17)	0.004
**eGFR, ml/min/1.73m** ^**2**^					
Continuous variable per unit		0.984 (0.983–0.985)	<0.001	0.990 (0.989–0.992)	<0.001
Categories					
≥60	4556 (10.95%)	ref		ref	
<60	2186 (22.12%)	2.07 (1.96–2.18)	<0.001	1.49 (1.41–1.58)	<0.001

*The lipoprotein(a) model adjusted for age, gender, percutaneous coronary intervention; acute myocardial infarction; hypertension; diabetes mellitus; anemia; stroke; congestive heart failure; coronary artery disease; valvular heart disease; atrial fibrillation; low-density lipoprotein cholesterol; high-density lipoprotein cholesterol; triglycerides; chronic kidney disease; angiotensin-converting enzyme inhibitor or angiotensin receptor blocker; beta-blockers; statins*.

*The eGFR model adjusted for age, gender, percutaneous coronary intervention; acute myocardial infarction; hypertension; diabetes mellitus; anemia; stroke; congestive heart failure; coronary artery disease; valvular heart disease; atrial fibrillation; low-density lipoprotein cholesterol; high-density lipoprotein cholesterol; triglycerides; lipoprotein(a); angiotensin-converting enzyme inhibitor or angiotensin receptor blocker; beta-blockers; statins*.

**Figure 1:**
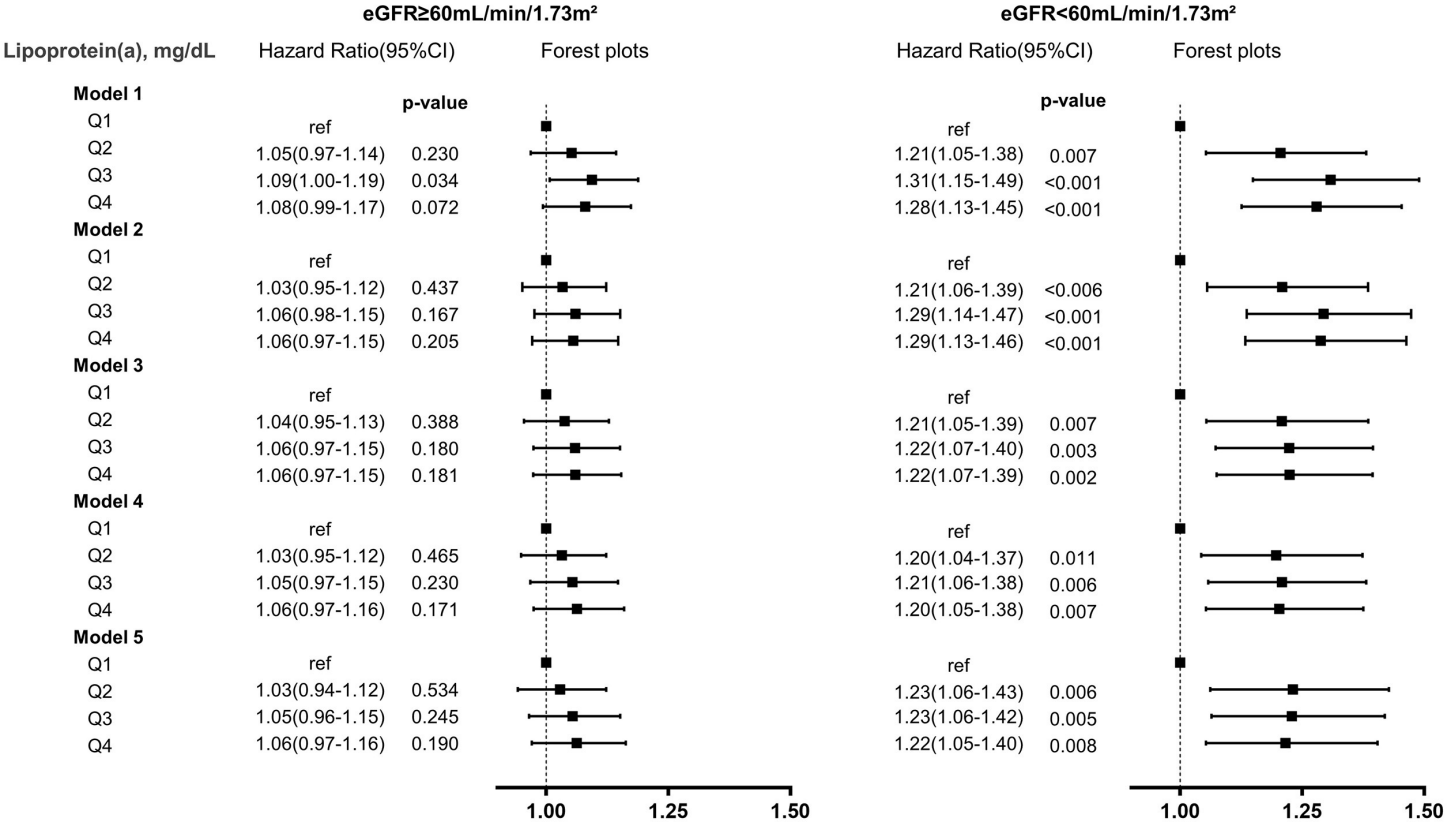
Lipoprotein(a)-associated risk of all-cause mortality in different eGFR levels. model (1) unadjusted; (2) adjusted for demographic characteristics (gender and age); (3) adjusted for demographic characteristics, medical history (percutaneous coronary intervention, acute myocardial infarction, hypertension, diabetes mellitus, anemia, stroke, congestive heart failure, coronary artery disease, valvular heart disease, atrial fibrillation); (4) adjusted for demographic characteristics, medical history, and laboratory tests (low-density lipoprotein cholesterol, high-density lipoprotein cholesterol, triglycerides); (5) adjusted for demographic characteristics, medical history, laboratory tests, and medications (angiotensin-converting enzyme inhibitor or angiotensin receptor blocker, beta-blockers, statins).

**Figure 2:**
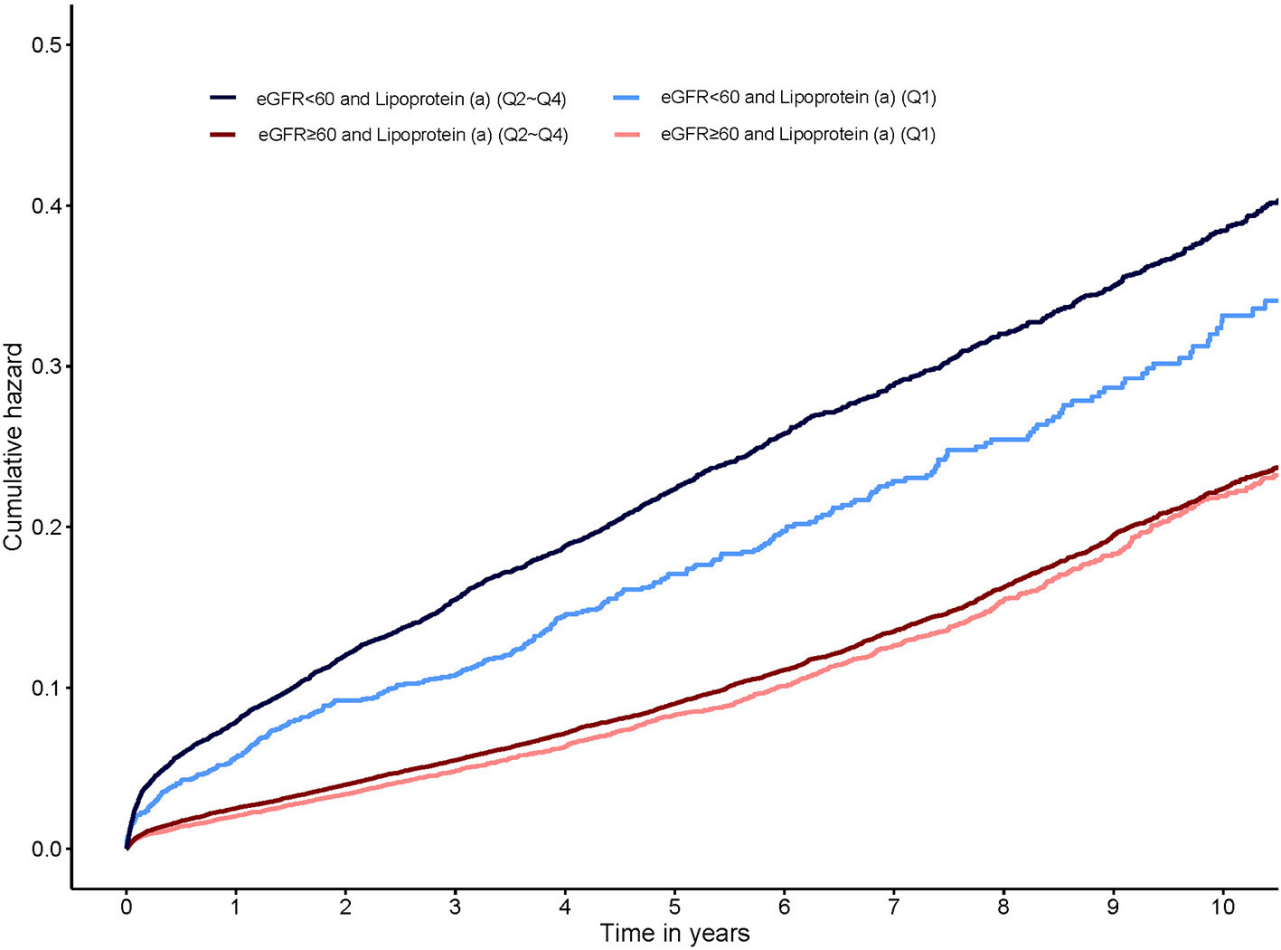
Kaplan-Meier curves for cumulative hazard of all-cause mortality by lipoprotein(a) categories stratified eGFR.

In patients with eGFR <60 mL/min/1.73m^2^, lipoprotein(a) Q2, Q3, and Q4 all had a 23% increased adjusted risk of death relative to Q1 (All of *p* <0.05). In patients with eGFR ≥60 mL/min/1.73m^2^, there were not significantly differences in adjusted risk of death between lipoprotein(a) Q2, Q3 as well as Q4 and Q1, respectively (Figure 1). In the subsequent analyses, lipoprotein(a) was dichotomized into low-risk group (Q1) and high-risk group (Q2–Q4). The cumulative hazard for all-cause mortality across lipoprotein(a) (Q1 vs. Q2–Q4) and eGFR categories shown in Figure 2. Compared with lipoprotein(a) Q1, lipoprotein(a) Q2–Q4 was strongly associated with increased adjusted risk of death in patients with eGFR <60 mL/min/1.73m^2^ (HR: 1.23 [95% CI: 1.08–1.39]), while such association was not significant in patients with eGFR ≥60 mL/min/1.73m^2^ (HR: 1.05 [95% CI: 0.97–1.13]). P for interaction between lipoprotein(a) (Q1 vs. Q2–Q4) and eGFR (≥60 vs. eGFR <60 mL/min/1.73m^2^) on all-cause mortality was 0.019 (Table 3). More information on confounding variables of Table 3 can be detailed in Supplementary Table 3.

**Table 3:** HR of all-cause mortality across lipoprotein(a) categories in eGFR ≥60 mL/min/1.73m^2^ and eGFR <60 mL/min/1.73m^2^.

**Categories**		**Crude**		**Adjusted**	
	**Events (%)**	**HR (95% CI)**	***P*-value**	**HR (95% CI)**	***P*-value**
**eGFR** **≥60 mL/min/1.73m**^**2**^					
Lipoprotein(a)Q1	1074 (9.87%)	ref		ref	
Lipoprotein(a)Q2-Q4	3482 (11.33%)	1.09 (1.01–1.16)	0.019	1.05 (0.97–1.13)	0.224
**eGFR** ** <60 mL/min/1.73m**^**2**^					
Lipoprotein(a)Q1	355 (18.35%)	ref		ref	
Lipoprotein(a)Q2-Q4	1831 (23.04%)	2.29 (2.13–2.47)	<0.001	1.23 (1.08–1.39)	0.002
^*^P for interaction		<0.001		0.019	

* *p-value for interaction test: 2-way interaction of lipoprotein(a) (Q1 vs. Q2–Q4) and renal function groups (eGFR ≥60 mL/min/1.73m^2^ vs. eGFR <60 mL/min/1.73m^2^)*.

*Adjusted for age, gender, percutaneous coronary intervention; acute myocardial infarction; hypertension; diabetes mellitus; anemia; stroke; congestive heart failure; coronary artery disease; valvular heart disease; atrial fibrillation; low-density lipoprotein cholesterol; high-density lipoprotein cholesterol; triglycerides; angiotensin-converting enzyme inhibitor or angiotensin receptor blocker; beta-blockers; statins*.

## Discussions

To our knowledge, this is the first large cohort real-world study to demonstrate the relationship between baseline eGFR and lipoprotein(a)-associated risk of all-cause mortality in patients undergoing CAG. Our main finding is that lipoprotein(a) has an eGFR- related modifier effect on all-cause mortality: higher lipoprotein(a) concentration has higher mortality in patients undergoing CAG with eGFR <60 mL/min/1.73m^2^, but this effect was not significant in patients eGFR ≥60 mL/min/1.73m^2^.

In the present study, elevated lipoprotein(a) level exhibited significant associations with the increased risk of all-cause mortality after correcting potentially confounding variables. In recent years, with the rapid development of medical technology and the rise of intervention of lipoprotein(a), baseline lipoprotein(a) has become the focus of research again. Many studies have proposed increased plasma lipoprotein(a) levels as an independent risk factor for long-term cardiovascular adverse events (18–21). In fact, lipoprotein(a) is an inherited atherogenic lipoprotein, and more than 90% of the variance in concentrations can be explained by genetics (22). The synthesis and metabolism of lipoprotein(a) is independent with other lipoproteins or lipid components. Theoretically, there is no significant interaction between lipoprotein(a) and other lipoproteins or lipid. In addition, other lipoproteins containing LDL-C, HDL-C and TRIG have been recognized as associated with prognosis. Statins used to lower LDL-C may increase plasma levels of the highly atherogenic molecule lipoprotein(a) (23). Considering the potential effects of lipoproteins and statins, our study has adjusted for those potential confounders. The possible underlying mechanism may be lipoprotein(a) potentially constitutes a molecular link between the processes of atherosclerosis (mediated by the LDL-like moiety) and thrombosis [mediated by the apo(a) moiety] that together precipitate events such as myocardial infarction and ischemic stroke (24, 25).

Renal function deteriorates, lipoprotein(a) concentrations increase. The association between kidney function and lipoprotein(a) levels has received interest since the initial observation of elevated lipoprotein(a) levels among dialysis patients in the end of 20th century (26). Previous studies showed the association of eGFR and lipoprotein(a) that higher lipoprotein(a) levels have been observed with reduced eGFR, even in the earliest stages of renal impairment (24, 27–31). Our results also show that there is an inverse correlation between lipoprotein(a) and eGFR in patients undergoing CAG. The possible underlying mechanism may be that elevated plasmatic lipoprotein(a) levels in nephrotic patients could be linked to increased synthesis of proteins in the liver as physiological reaction to proteinuria (3, 12, 23). The another potential hypotheses is that impaired renal function may lead to the decrease of the metabolic clearance rate, thus increasing the concentration of lipoprotein(a) (32).

It should be highlighted that a higher level of lipoprotein(a) in patients with poorer renal function tended to have a higher risk of all-cause mortality. In our study, the patients with relatively good renal function, elevated lipoprotein(a) had no significant risk of all-cause mortality, while the individuals with eGFR <60, the higher level of lipoprotein(a) was associated with a sharp increased risk of all-cause mortality. Few studies have reported the association of eGFR and lipoprotein(a) and all-cause mortality. Recently, Xu et al. (18) found that high lipoprotein(a) value was associated with the occurrence of death in 427 consecutive patients who underwent PCI with CKD. Konishi et al. (19) also demonstrated that high lipoprotein(a) levels were associated with a composite of all-cause mortality and incident acute coronary syndrome in a cohort of 904 patients with CKD among 3,508 patients who underwent the first PCI. However, studies on lipoprotein(a)-related risk of death focus on patients with poor renal function, the regulatory effect of eGFR stratification on lipoprotein(a)-associated mortality has not been adequately addressed. Our study showed that all-cause mortality is significantly increased in lipoprotein(a) (Q2–Q4) group compared with lipoprotein(a) Q1 in eGFR <60 mL/min/1.73m^2^, a difference not observed in eGFR ≥60 mL/min/1.73m^2^. This finding may guide clinicians to develop the optimal individualized treatment strategy. The possible mechanism is that elevated lipoprotein (a) may contribute to the acceleration of glomerular injury in various renal diseases by inducing activation of reactive oxygen metabolites (33), and decreased renal catabolism may lead to accumulation of lipoprotein (a) and thus increased concentration (28), resulting in a vicious cycle of accelerated death.

All those findings strongly support the need for physicians to practice early risk stratification according to the level of lipoprotein(a) and eGFR in hospitalization. Clinicians should stay abreast of the current scientific evidence to provide the most meaningful and effective lipid metabolism control, aiming to individualize anti-atherosclerosis support. Although, lipoprotein(a) level is under strong genetic control and not susceptible to nutrition or physical activity (22, 34). Fortunately, several novel modalities of therapeutic interventions to lower plasma lipoprotein(a) concentrations have been shown. A clear effect of anti-atherosclerosis on lipoprotein(a)is exerted by protein convertase subtilisin/kexin type 9 (PCSK9) antagonists (35, 36). Currently, emerging therapies antisense oligonucleotides targeting apo(a) could potently reduce plasma lipoprotein(a) (37); However, these new drugs are too expensive to be universally available in a developing country. Meanwhile, reducing lipoprotein(a) concentration may not be that imperative in patients with eGFR≥60 mL/min/1.73m^2^. It needs to be stressed to conduct clinical intensive management and the lipid-lowering therapy in patients with worse renal function and higher level of lipoprotein(a). Further studies are needed to prospectively evaluate the efficacy of lipoprotein(a) metabolism control on outcomes in patients with lower and higher levels of eGFR.

Limitations of this study should be considered. First, because it was a single-center, retrospective study, our inferences did not reflect direct causality. We must always recognize the potential for residual, uncontrolled confounding that might partly explain the associations. Secondly, there might be restrictions regarding generalization across ethnicities as we only included Chinese individuals; however, we are not aware of any data that the present results should not apply to people of most ethnicities. Thirdly, the sample size between lipoprotein(a) Q1 and lipoprotein(a) Q2–Q4 was indeed unequal. However, the large sample size can make up for this imbalance which may not affect the statistic validity. Forth, given that the eGFR and lipoprotein(a) concentration were conducted only at a single time point, we did not analyze the changes in lipoprotein(a) and renal status over time and their interaction with all-cause mortality. Fifth, there was limited data on the included patients, without information about proteinuria and albuminuria, which might help us to better understand the relationship between kidney function and lipoprotein(a). Last, although we established that eGFR might help classify risk in lipoprotein(a) categories, due to the observational nature of our study, prospective clinical trials are needed to establish whether aggressive lipoprotein(a) management in these groups will improve long-term outcomes.

## Conclusions

Elevated lipoprotein(a) was associated with increased risk of all-cause mortality and such an association was modified by the baseline eGFR in CAG patients. More attention should be paid to the patients with reduced eGFR and elevated lipoprotein(a), and the appropriate lipoprotein(a) intervention is required. Future studies are needed to explore the potential mechanism of the association of lipoprotein(a) with all-cause mortality across categories of eGFR.

## Data Availability Statement

The original contributions presented in the study are included in the article/Supplementary Material, further inquiries can be directed to the corresponding authors.

## Ethics Statement

All traceable personal identifiers were removed from the analytic dataset to protect patients' privacy. The study protocol was approved by Guangdong Provincial People's Hospital ethics committee and the study was performed according to the declaration of Helsinki.

## Author Contributions

ZDH, JLiu, SC, and LC: research idea and study design. ZDH, JLu, YYa, JLia, YH, YYu, HH, QL, BW, SL, ZY, DX, YL, KC, ZGH, and JN: data acquisition. JLiu and LC: data analysis/interpretation. ZDH and SL: statistical analysis. SC and LC: supervision and mentorship. LC: writing guidance. All authors contributed important intellectual content during manuscript drafting or revision and accepts accountability for the overall work by ensuring that questions on the accuracy or integrity of any portion of the work are appropriately investigated and resolved, and read and approved the final version.

## Funding

This research was funded and supported by Fujian Province Natural Science Foundation (Grant number: 2019J01617), Beijing Lisheng Cardiovascular Health Foundation (No. LHJJ20141751), Study on the function and mechanism of the potential target for early warning of cardiorenal syndrome after acute myocardial infarction based on transformism (DFJH201919), Natural Science Foundation of Guangdong Province General Project (2020A1515010940), and Guangdong Provincial Key Laboratory of Coronary Heart Disease Prevention (2017B030314041). The funders had no role in the study design, data collection, and analysis, decision to publish, or preparation of the manuscript.

## Conflict of Interest

The authors declare that the research was conducted in the absence of any commercial or financial relationships that could be construed as a potential conflict of interest.

## Publisher's Note

All claims expressed in this article are solely those of the authors and do not necessarily represent those of their affiliated organizations, or those of the publisher, the editors and the reviewers. Any product that may be evaluated in this article, or claim that may be made by its manufacturer, is not guaranteed or endorsed by the publisher.
