# Supplementary material for: Association of Lipoprotein(a)-Associated Mortality and the Estimated Glomerular Filtration Rate Level in Patients Undergoing Coronary Angiography: A 51,500 Cohort Study
Source: Front Cardiovasc Med. 2021 Nov 17;8:747120. doi: 10.3389/fcvm.2021.747120 (PMC8635642; doi:10.3389/fcvm.2021.747120)

Supplementary Material

##
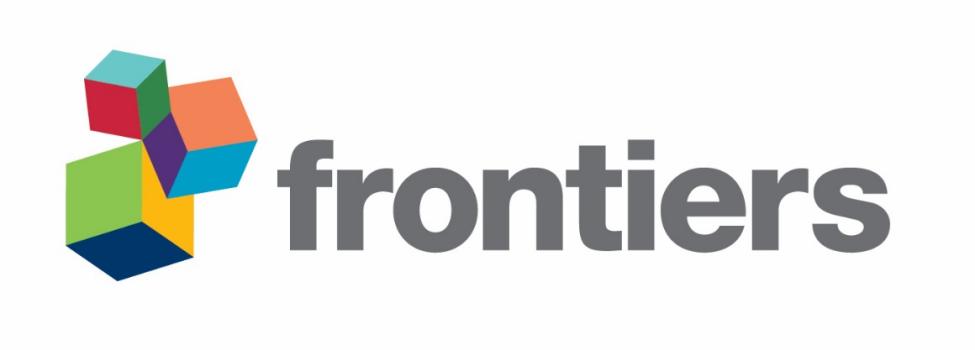


## Supplementary Table 1. Baseline characteristics across lipoprotein(a) (Q1, Q2, Q3 and Q4) and eGFR categories

| **Characteristics** | **eGFR≥60, Q1** | **eGFR<60, Q1** | **eGFR≥60, Q2** | **eGFR<60, Q2** | **eGFR≥60, Q3** | **eGFR<60, Q3** | **eGFR≥60, Q4** | **eGFR<60, Q4** |
| --- | --- | --- | --- | --- | --- | --- | --- | --- |
|  | n=10883 | n=1935 | n=10598 | n=2283 | n=10241 | n=2674 | n=9897 | n=2989 |
| **Demographic characteristics** | | | | | | | | |
| Age, year | 60.3±10.3 | 68.1±9.7 | 61.0±10.4 | 67.9±10.0 | 61.6±10.2 | 68.3±10.0 | 61.1±10.2 | 67.6±9.6 |
| Female, n (%) | 3456 (31.8) | 674 (34.8) | 3348 (31.6) | 769 (33.7) | 3046 (29.7) | 852 (31.9) | 2934 (29.6) | 1033 (34.6) |
| **Medical history** | | | | | | | | |
| AMI, n (%) | 922 (8.5) | 230 (11.9) | 1378 (13.0) | 402 (17.6) | 1675 (16.4) | 515 (19.3) | 1758 (17.8) | 597 (20.0) |
| CHF, n (%) | 827 (7.6) | 300 (15.5) | 896 (8.5) | 425 (18.6) | 944 (9.2) | 581 (21.8) | 889 (9.0) | 631 (21.2) |
| Anemia, n (%) | 2492 (23.1) | 865 (45.0) | 2640 (25.2) | 999 (44.1) | 2793 (27.5) | 1312 (49.5) | 2925 (29.8) | 1607 (54.2) |
| HT, n (%) | 5203 (47.9) | 1325 (68.6) | 4927 (46.5) | 1533 (67.3) | 4818 (47.1) | 1761 (65.9) | 4711 (47.7) | 2067 (69.4) |
| DM, n (%) | 2492 (22.9) | 660 (34.2) | 2147 (20.3) | 697 (30.6) | 2135 (20.9) | 862 (32.3) | 2052 (20.8) | 981 (32.9) |
| PCI, n (%) | 4297 (39.5) | 949 (49.0) | 4795 (45.2) | 1223 (53.6) | 5165 (50.4) | 1529 (57.2) | 5756 (58.2) | 1898 (63.5) |
| CAD, n (%) | 6328 (58.2) | 6681 (63.0) | 6962 (68.0) | 7478 (75.6) | 1365 (70.5) | 1677 (73.5) | 2069 (77.4) | 2490 (83.3) |
| VHD, n (%) | 2075 (19.1) | 381 (19.7) | 1872 (17.7) | 432 (19.0) | 1694 (16.6) | 497 (18.6) | 1322 (13.4) | 446 (15.0) |
| AF, n (%) | 1077 (9.9) | 251 (13.0) | 904 (8.5) | 268 (11.7) | 762 (7.4) | 296 (11.1) | 549 (5.5) | 211 (7.1) |
| **Laboratory tests** | | | | | | | | |
| WBC, 10^9^/L | 7.44±2.33 | 8.20±3.35 | 7.65±2.58 | 8.34±3.18 | 7.74±2.53 | 8.48±3.26 | 7.78±2.49 | 8.45±3.15 |
| HGB, g/L | 135.87±15.41 | 128.26±18.94 | 135.18±15.21 | 127.93±19.85 | 134.28±15.78 | 125.76±20.75 | 133.58±15.43 | 122.50±20.83 |
| TC, mmol/L | 4.35±1.11 | 4.23±1.13 | 4.54±1.11 | 4.38±1.15 | 4.65±1.15 | 4.52±1.18 | 4.80±1.23 | 4.69±1.26 |
| TRIG, mmol/L | 1.82±1.55 | 1.94±1.62 | 1.57±1.11 | 1.68±1.34 | 1.46±0.90 | 1.56±1.00 | 1.49±0.88 | 1.56±0.87 |
| APOA, g/L | 1.16±0.27 | 1.10±0.27 | 1.14±0.27 | 1.09±0.26 | 1.13±0.28 | 1.08±0.26 | 1.12±0.28 | 1.07±0.27 |
| APOB, g/L | 0.79±0.22 | 0.77±0.22 | 0.85±0.22 | 0.82±0.22 | 0.87±0.24 | 0.86±0.24 | 0.91±0.24 | 0.91±0.25 |
| LDLC, mmol/L | 2.58±0.86 | 2.44±0.88 | 2.79±0.90 | 2.63±0.89 | 2.91±0.94 | 2.79±0.92 | 3.05±0.99 | 2.94±1.01 |
| HDLC, mmol/L | 1.03±0.27 | 0.99±0.29 | 1.04±0.27 | 0.99±0.27 | 1.04±0.27 | 1.00±0.27 | 1.05±0.28 | 1.00±0.28 |
| HbA1c, % | 6.32±1.27 | 6.62±1.35 | 6.29±1.22 | 6.54±1.20 | 6.32±1.27 | 6.60±1.36 | 6.39±1.37 | 6.66±1.39 |
| **Medications** | | | | | | | | |
| ACEI or ARB, n (%) | 3820 (36.3) | 699 (37.8) | 4090 (39.7) | 866 (39.7) | 4163 (41.7) | 1016 (39.7) | 4355 (44.9) | 1198 (42.0) |
| Beta-blockers, n (%) | 6860 (65.3) | 1334 (72.2) | 6909 (67.1) | 1547 (71.0) | 6933 (69.4) | 1819 (71.1) | 7103 (73.2) | 2171 (76.0) |
| Statins, n (%) | 7672 (73.0) | 1444 (78.1) | 7817 (75.9) | 1734 (79.6) | 7852 (78.6) | 2057 (80.4) | 8125 (83.7) | 2470 (86.5) |

Values are, n (%) or mean ± SD.

Abbreviations: AMI, acute myocardial infarction; CHF, congestive heart failure; HT, hypertension; DM, diabetes mellitus; PCI, percutaneous coronary intervention; CAD, coronary artery disease; VHD, valvular heart disease; AF, atrial fibrillation; WBC, white blood cell; HGB, hemoglobin; TC, serum total cholesterol; TRIG, triglycerides; APOA, apolipoprotein A; APOB, apolipoprotein B; LDL-C, low-density lipoprotein cholesterol; HDL-C, high-density lipoprotein cholesterol; HbA1c, glycosylated hemoglobin; ACEI or ARB, angiotensin-converting enzyme inhibitor or angiotensin receptor blocker.

**Supplementary Table 2.** Baseline characteristics across Lipoprotein(a) (Q1, Q2~Q4) and eGFR categories

| **Characteristics** | **eGFR≥60, Q1** | **eGFR<60, Q1** | **P value** | **eGFR≥60, Q2~Q4** | **eGFR<60, Q2~Q4** | | **P value** |
| --- | --- | --- | --- | --- | --- | --- | --- |
|  | n=10883 | n=1935 |  | n=30736 | n=7946 | |  |
| **Demographic characteristics** | | | | | | | |
| Age, year | 60.3±10.3 | 68.1±9.7 | <0.001 | 61.2±10.3 | 67.9±9.9 | 0.579 | |
| Female, n (%) | 3456 (31.8) | 674 (34.8) | 0.006 | 9328 (30.3) | 2654 (33.4) | 0.243 | |
| **Medical history** | | | | | | | |
| AMI, n (%) | 922 (8.5) | 230 (11.9) | <0.001 | 4811 (15.7) | 1514 (19.1) | <0.001 | |
| CHF, n (%) | 827 (7.6) | 300 (15.5) | <0.001 | 2729 (8.9) | 1637 (20.6) | <0.001 | |
| Anemia, n (%) | 2492 (23.1) | 865 (45.0) | <0.001 | 8358 (27.4) | 3918 (49.7) | <0.001 | |
| Hypertension, n (%) | 5203 (47.9) | 1325 (68.6) | 0.167 | 14456 (47.1) | 5361 (67.6) | 0.425 | |
| DM, n (%) | 2492 (22.9) | 660 (34.2) | <0.001 | 6334 (20.6) | 2540 (32.0) | 0.077 | |
| PCI, n (%) | 4297 (39.5) | 949 (49.0) | <0.001 | 15716 (51.1) | 4650 (58.5) | <0.001 | |
| CAD, n (%) | 6328 (58.2) | 1365 (70.5) | <0.001 | 21121 (68.7) | 6236 (78.5) | <0.001 | |
| VHD, n (%) | 2075 (19.1) | 381 (19.7) | <0.001 | 4888 (15.9) | 1375 (17.3) | 0.016 | |
| AF, n (%) | 1077 (9.9) | 251 (13.0) | <0.001 | 2215 (7.2) | 775 (9.8) | <0.001 | |
| **Laboratory tests** | | | | | | | |
| WBC, 10^9^/L | 7.44±2.33 | 8.20±3.35 | <0.001 | 7.72±2.54 | 8.43±3.20 | 0.005 | |
| HGB, g/L | 135.87±15.41 | 128.26±18.94 | <0.001 | 134.37±15.48 | 125.16±20.64 | <0.001 | |
| TC, mmol/L | 4.35±1.11 | 4.23±1.13 | <0.001 | 4.66±1.17 | 4.55±1.21 | <0.001 | |
| TRIG, mmol/L | 1.82±1.55 | 1.94±1.62 | <0.001 | 1.51±0.97 | 1.59±1.07 | <0.001 | |
| APOA, g/L | 1.16±0.27 | 1.10±0.27 | <0.001 | 1.13±0.28 | 1.08±0.26 | <0.001 | |
| APOB, g/L | 0.79±0.22 | 0.77±0.22 | <0.001 | 0.88±0.23 | 0.87±0.24 | <0.001 | |
| LDL-C, mmol/L | 2.58±0.86 | 2.44±0.88 | <0.001 | 2.91±0.95 | 2.80±0.96 | <0.001 | |
| HDL-C, mmol/L | 1.03±0.27 | 0.99±0.29 | <0.001 | 1.04±0.28 | 0.99±0.27 | 0.240 | |
| HbA1c, % | 6.32 (1.27) | 6.62 (1.35) | 0.413 | 6.33 (1.28) | 6.61 (1.33) | 0.693 | |
| **Medications** | | | | | | | |
| ACEI or ARB, n (%) | 3820 (36.3) | 699 (37.8) | <0.001 | 12608 (42.0) | 3080 (40.6) | 0.034 | |
| Beta-blockers, n (%) | 6860 (65.3) | 1334 (72.2) | <0.001 | 20945 (69.8) | 5537 (72.9) | 0.548 | |
| Statins, n (%) | 7672 (73.0) | 1444 (78.1) | <0.001 | 23794 (79.3) | 6261 (82.4) | <0.001 | |

Values are, n(%) or mean ± SD.

Abbreviation: eGFR, estimated glomerular filtration rate; AMI, acute myocardial infarction; CHF, congestive heart failure; DM, diabetes mellitus; PCI, percutaneous coronary intervention; CAD, coronary artery disease; VHD, valvular heart disease; AF, atrial fibrillation; WBC, white blood cell; HGB, hemoglobin; TC, serum total cholesterol; TRIG, triglycerides; APOA, apolipoprotein A; APOB, apolipoprotein B; LDL-C, low-density lipoprotein cholesterol; HDL-C, high-density lipoprotein cholesterol; HbA1c, glycosylated hemoglobin; ACEI or ARB, angiotensin-converting enzyme inhibitor or angiotensin receptor blocker

**Supplementary Table 3.** Multivariate Cox proportional hazards model for the association between lipoprotein(a) and all-cause death in eGFR ≥60 mL/min/1.73m² and eGFR <60 mL/min/1.73m²

|  | **eGFR ≥60 mL/min/1.73m²** | |  | **eGFR <60 mL/min/1.73m²** | |
| --- | --- | --- | --- | --- | --- |
|  | **HR (95% CI)** | **P value** |  | **HR (95% CI)** | **P value** |
| lipoprotein(a)  Q2-Q4 vs. Q1 | 1.05 (0.97-1.13) | 0.224 |  | 1.23 (1.08-1.39) | 0.001 |
| age, year | 1.02 (1.02-1.02) | <0.001 |  | 1.02 (1.01-1.02) | <0.001 |
| gender | 0.80 (0.74-0.86) | <0.001 |  | 0.83 (0.75-0.92) | <0.001 |
| PCI | 0.90 (0.82-0.97) | 0.001 |  | 0.84 (0.75-0.95) | 0.004 |
| AMI | 1.06 (0.96-1.17) | 0.259 |  | 1.03 (0.91-1.17) | 0.649 |
| HT | 1.01 (0.94-1.08) | 0.795 |  | 0.98 (0.88-1.09) | 0.705 |
| DM | 1.10 (1.02-1.19) | 0.012 |  | 1.23 (1.11-1.36) | <0.001 |
| Anemia | 1.20 (1.13-1.29) | <0.001 |  | 1.39 (1.26-1.53) | <0.001 |
| Stroke | 1.22 (1.07-1.39) | 0.003 |  | 1.02 (0.87-1.20) | 0.812 |
| CHF | 1.41 (1.27-1.58) | <0.001 |  | 1.91 (1.71-2.13) | <0.001 |
| CAD | 1.21 (1.10-1.34) | <0.001 |  | 1.28 (1.09-1.50) | 0.003 |
| VHD | 1.11 (1.00-1.23) | 0.049 |  | 1.22 (1.06-1.40) | 0.007 |
| AF | 1.11 (0.98-1.25) | 0.099 |  | 1.04 (0.88-1.22) | 0.679 |
| LDLC, mmol/L | 0.98 (0.95-1.01) | 0.251 |  | 1.05 (1.00-1.11) | 0.053 |
| HDLC, mmol/L | 0.90 (0.80-1.01) | 0.071 |  | 0.71 (0.59-0.85) | <0.001 |
| TRIG, mmol/L | 0.95 (0.91-0.98) | 0.001 |  | 0.98 (0.93-1.03) | 0.366 |
| Statins | 0.76 (0.69-0.84) | <0.001 |  | 0.91 (0.78-1.06) | 0.208 |
| ACEI or ARB | 0.99 (0.92-1.05) | 0.698 |  | 0.88 (0.80-0.97) | 0.01 |
| Beta-blockers | 0.98 (0.91-1.06) | 0.619 |  | 1.02 (0.91-1.13) | 0.750 |

**Abbreviation:** PCI, percutaneous coronary intervention; AMI, acute myocardial infarction; HT, hypertension; DM, diabetes mellitus; CHF, congestive heart failure; CAD, coronary artery disease; VHD, valvular heart disease; AF, atrial fibrillation; LDL-C, low-density lipoprotein cholesterol; HDL-C, high-density lipoprotein cholesterol; TRIG, triglycerides; ACEI or ARB, angiotensin-converting enzyme inhibitor or angiotensin receptor blocker.

**Supplementary Figure 1.** Patient flow diagram

**
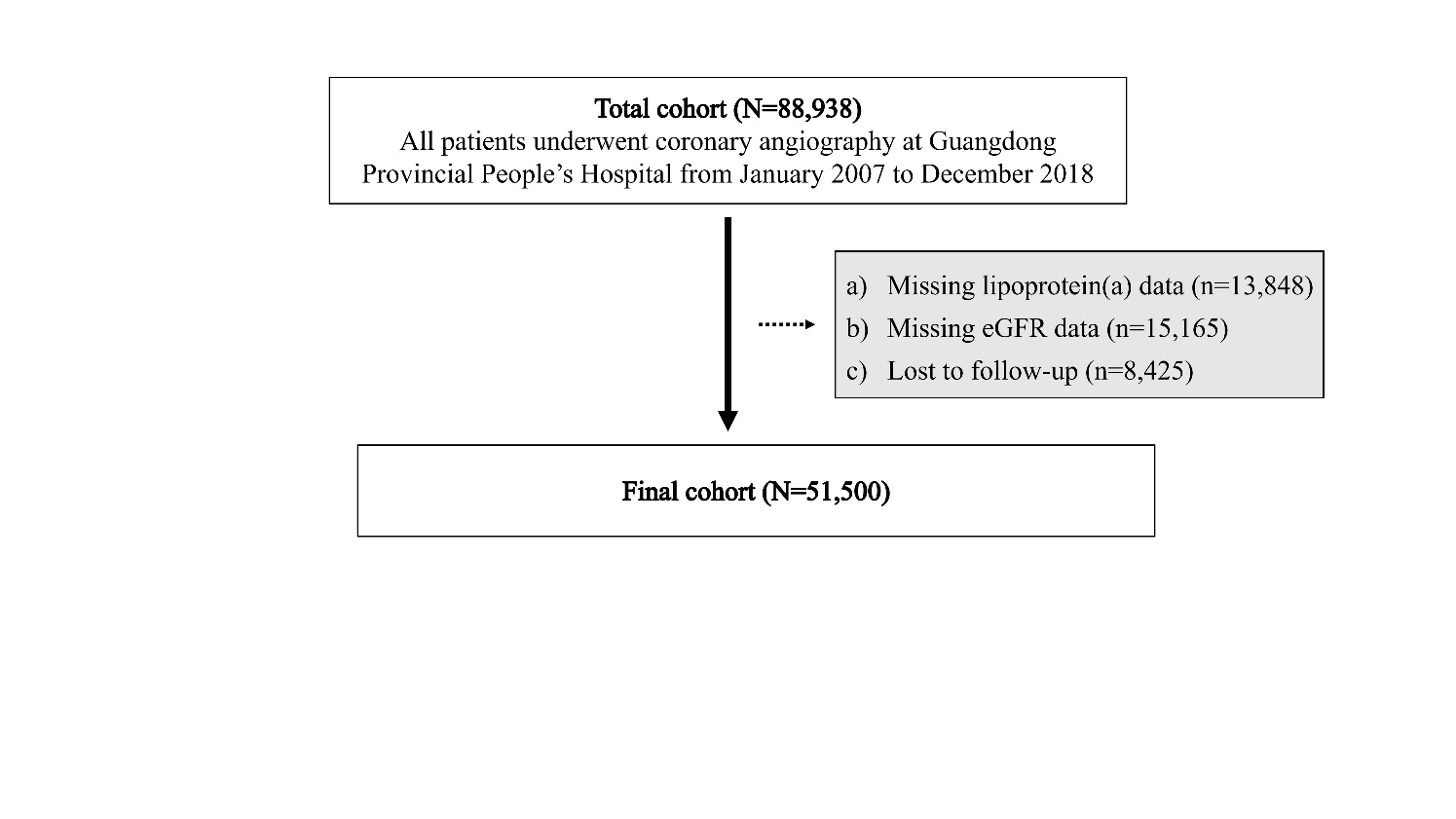
**

**Supplement Figure 2.** Lipoprotein(a) concentrations according to different categories of eGFR

**
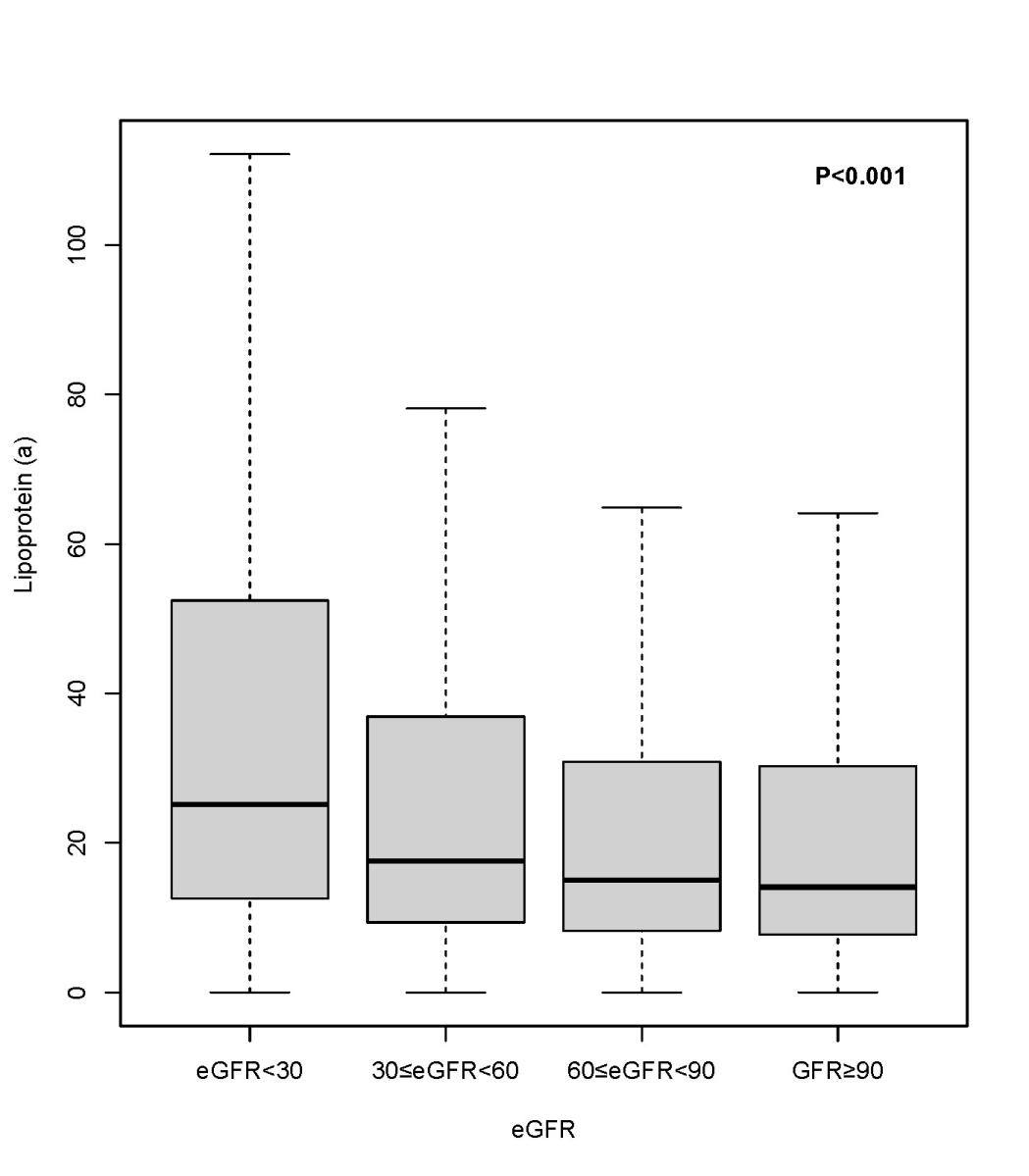
**

**Note:** The differences of characteristics in box plot were compared using Kruskal-Wallis.

**Supplement Figure 3.** Kaplan-Meier curves for cumulative hazard of all-cause mortality stratified by lipoprotein(a) categories and eGFR level.


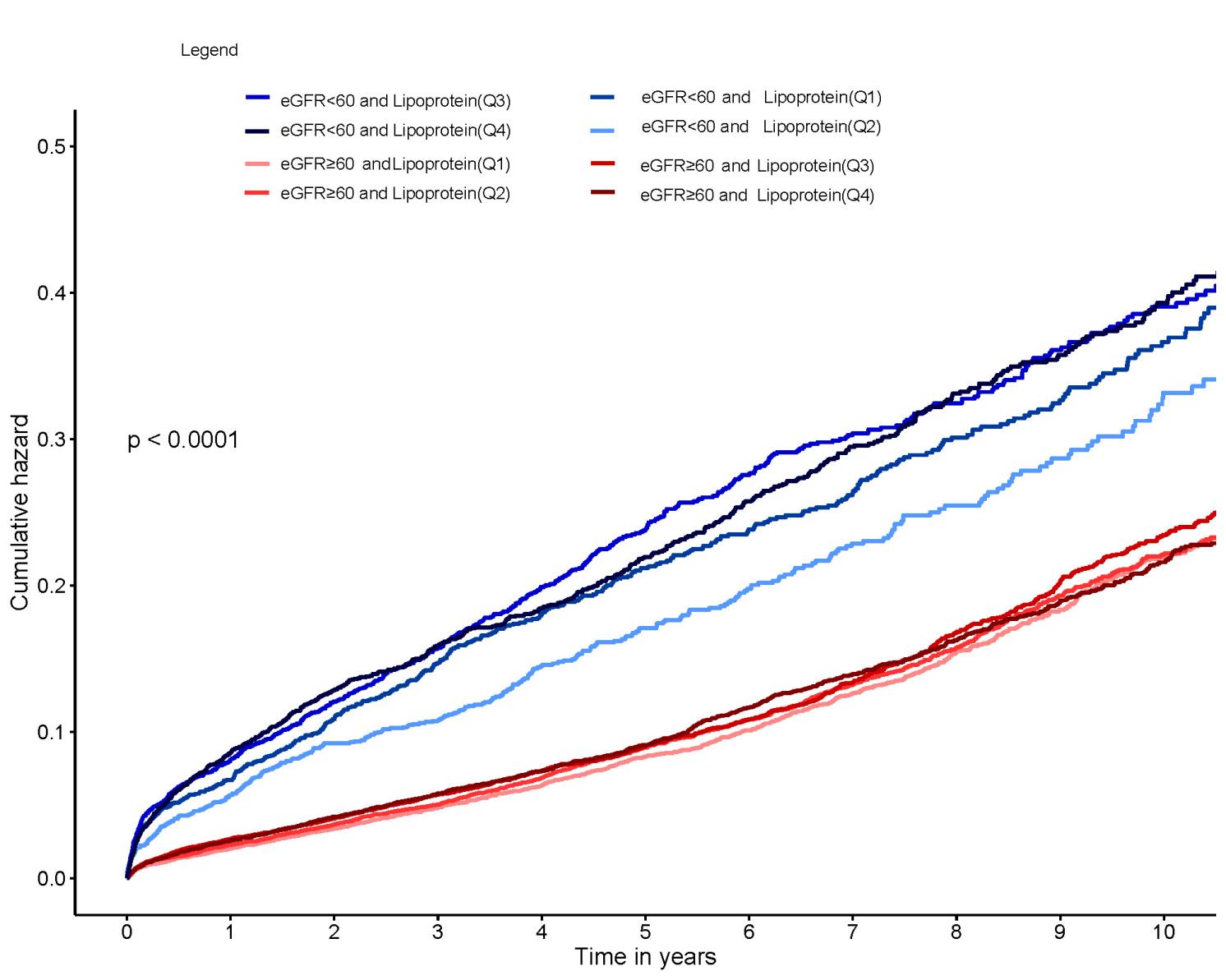

Supplement: Supplementary file 1 [file Data_Sheet_1.docx]
